# Validation of urinary biomarkers for accurate diagnosis of urinary tract infections in older adults across primary care, hospitals and long-term care facilities in the Netherlands and UK (UTI-GOLD): a multicentre observational study protocol

**DOI:** 10.1136/bmjopen-2025-103311

**Published:** 2025-06-18

**Authors:** Nora El Moussaoui, Esther van Andel, Martha T van der Beek, Jessica A Vlot, Manu P Bilsen, Cees van Nieuwkoop, Fanny N Lauw, Nathalie M Delfos, Janneke E Stalenhoef, Martijn Sijbom, Abimbola A Akintola, Wilco P Achterberg, Jelle J Goeman, Eliane M S Leyten, Janneke I M van Uhm, Paul L A M Corstjens, Simon P Mooijaart, Simon P Conroy, Christa M Cobbaert, Leo G Visser, Merel M C Lambregts

**Affiliations:** 1 Leiden University Center of Infectious Diseases (LUCID), Leiden University Medical Center, Leiden, Netherlands; 2 Department of Clinical Chemistry and Laboratory Medicine, Leiden University Medical Center, Leiden, Netherlands; 3 Department of Internal Medicine, HagaZiekenhuis, Den Haag, Netherlands; 4 Department of Public Health and Primary Care/ Campus The Hague, Leiden University Medical Center, The Hague, Netherlands; 5 Department of Internal Medicine, Alrijne Hospital, Leiderdorp, Netherlands; 6 Department of Internal Medicine, OLVG Locatie Oost, Amsterdam, Netherlands; 7 The university Network for the Care Sector South Holland, leiden, Netherlands; 8 Department of Biomedical Data Sciences, Leiden University Medical Center, Leiden, Netherlands; 9 Department of Internal Medicine, Haaglanden Medical Center, The Hague, Netherlands; 10 Department of Urology, Leiden University Medical Center, Leiden, Netherlands; 11 Department of Cell and Chemical Biology, Leiden University Medical Center, Leiden, ZH, Netherlands; 12 LUMC center for Medicine for Older People, Leiden University Medical Center, Leiden, Netherlands; 13 Medical Research Council Unit for lifelong Health and Ageing, University College London, London, UK

**Keywords:** Urinary tract infections, Infectious Diseases, Biomarkers

## Abstract

**Introduction:**

Urinary tract infections (UTIs) are highly prevalent and pose a significant burden among older adults. Accurate diagnosis in this population is challenging due to the high prevalence of pre-existing lower urinary tract symptoms, inability to express symptoms and asymptomatic bacteriuria. Current diagnostic tests are unreliable, often resulting in over- and underdiagnosis. A previous pilot study proposed a higher cut-off for pyuria and identified five promising biomarkers for the diagnosis of UTIs in older adults. The UTI-GOLD study aims to validate these five new biomarkers and the higher leucocyte cut-off as a diagnostic tool for UTIs in older people in a real-world setting.

**Methods and analysis:**

Between August 2024 and December 2027, an observational multicentre diagnostic accuracy study is being conducted across primary, secondary and tertiary healthcare facilities in the Netherlands and the UK. Adults ≥65 years with a suspected UTI will be considered eligible. Patients with pre-existing decision-making incapacity or an indwelling catheter will be excluded. UTI will be defined according to an international consensus-based reference standard. Biomarkers will be measured by liquid chromatography-mass spectrometry (neutrophil gelatinase-associated lipocalin, tissue inhibitor of metalloproteinase 2 and CXC motif chemokine ligand 9) and enzyme-linked immunosorbent assay (interleukin 6 and azurocidin). Pyuria will be quantified by automated microscopy and/or flow cytometry. Diagnostic accuracy measures will be calculated using the receiver operating characteristic curves, and sensitivity, specificity, likelihood ratios and predictive values will be reported for optimal cut-offs.

**Ethics and dissemination:**

The protocol was reviewed by the local Leiden University Medical Center research committee, who declared on 15 April 2024 that the medical research involving human subject act (Dutch abbreviation: WMO) does not apply to the current study (reference number nWMODIV2_2024025). The study also received approval from the NHS Research Ethics Committee in the UK (reference number 24/LO/0649).

The study findings will be published in a peer-reviewed journal, presented at academic congresses and shared with healthcare providers.

**Trial registration number:**

The study was registered at clinicaltrial.gov on the 24 September 2024 with registration number: NCT06610721.

**STRENGTHS AND LIMITATIONS**

- The broadly defined inclusion criteria and recruitment from different healthcare settings contribute to applicability in the real world.
- Participation does not require any extra hospital visits, enhancing the inclusivity of frail older people.
- To prevent selection bias, the study also accepts urine samples that are not collected according to the preferred clean catch (mid-stream) method.
- A research reference standard is used to define urinary tract infections, aiming to minimise the risk of misclassification that inevitably arises due to the atypical presentation of older adults and the lack of a golden standard.
- The exclusion of older people with pre-existing decision-making incapacity may restrict the generalisability of the study results.

## Introduction

 Urinary tract infections (UTIs) are one of the most common bacterial infections worldwide.^1^ Especially in older adults, UTIs are a significant cause of morbidity and mortality. UTIs substantially reduce quality of life,^2 3^ and UTI-related mortality rates increased notably world-wide during 1990 and 2019 for this age group.^4^ UTIs cause a significant clinical and economical strain on healthcare facilities.^5^ Despite the high prevalence and burden, diagnosing UTIs correctly presents significant challenges, especially in older adults. Older adults with comorbidities are prone to misdiagnosis of UTIs and in turn under- or overtreatment.^6^,^8^ The diagnosis of UTIs is based on a combination of symptomatology and urinalysis results, both of which are affected by age and multimorbidity.^9^ A major diagnostic difficulty in older patients is that symptoms of UTIs can be atypical or mimicked by comorbidities. Lower urinary tract symptoms (LUTS) such as urgency, urinary incontinence and frequency are common in older adults, but are often unrelated to UTI. These symptoms are frequently caused by age-associated comorbidities such as overactive bladder, vaginal atrophy or benign prostatic hyperplasia,^10^,^12^ resulting in a low specificity of lower urinary tract symptoms with increasing age. On the other hand, the sensitivity of lower urinary tract symptoms also decreases with age. Lower urinary tract symptoms can be absent in older people with UTI, including bacteraemic UTI.^11 12^ In addition, the higher prevalence of cognitive impairment (dementia or delirium) in this population may preclude a reliable identification of symptoms.

The second diagnostic challenge is the high prevalence of asymptomatic bacteriuria (ASB) in older people. Approximately 20%–50% of older women have ASB,^13^ of which 90% have concomitant pyuria.^14 15^ This phenomenon limits the diagnostic utility of the second key component of UTI diagnosis, pyuria and bacteriuria-based urinalysis, in this patient population. Previous research in older women showed that the current pyuria cut-off level of 10 cells per µL has a specificity of only 36%.^16^ The diagnostic challenge is even greater in primary care and long-term care facilities which often have only a limited diagnostic array, such as dipsticks available. This is problematic considering that the majority of UTIs are treated in these healthcare facilities. The current inaccuracy in diagnostic UTI workup can result in both over- and underdiagnosis of UTIs. Overdiagnosis drives inappropriate antibiotic use and the development of antimicrobial resistance, while underdiagnosis can result in delayed treatment, increased morbidity and mortality, and longer hospital stays.^17^

It is therefore crucial to improve diagnostic accuracy for UTI in older adults, particularly those with comorbidities and frailty.^7 18 19^ By providing reliable diagnostic markers together with appropriate cut-off values for UTIs in this population, suboptimal or delayed treatment can be avoided, which would significantly impact disease burden and improve patient outcomes. Multiple studies have identified useful biomarkers for the diagnosis of UTI.^20 21^ A recent study conducted by our group in a well-defined cohort of older women identified five promising markers for the diagnosis of UTIs. These include neutrophil gelatinase-associated lipocalin (NGAL), interleukin 6 (IL-6), azurocidin (AZU), tissue inhibitor of metalloproteinase 2 (TIMP2) and CXC motif chemokine ligand 9 (CXCL-9).^22^ NGAL is a protein that is produced by neutrophils and epithelial cells in the urinary tract and is a marker of injury and inflammation. IL-6 is a pro-inflammatory cytokine. AZU, a heparin-binding protein, is also released by neutrophils and has been identified as a marker of sepsis. TIMP-2 is a regulator of matrix metalloproteinases. CXCL9 is a chemokine induced by interferon gamma that is involved in the recruitment and activation of T cells and natural killer cells. These biomarkers hold significant potential for a new diagnostic test. In addition to these novel biomarkers, in the same cohort, the performance of an existing diagnostic tool: the urinary leucocyte (pyuria) count was investigated. The study showed that current cut-off values for pyuria lack specificity in older patients and proposed a higher, age-specific cut-off for the diagnosis of UTI. This age-specific cut-off could improve the performance of currently available diagnostic tests by tailoring their interpretation to the specific needs of older patients.

The UTI-GOLD study aims to validate five new biomarkers and the higher cut-off value for pyuria for UTI diagnosis in older patients in different real-world healthcare settings.

## Methods

### Study design and setting

The UTI-GOLD is an investigator-initiated observational multicentre, diagnostic accuracy study. Subjects will be enrolled at hospitals (n=6, the Netherlands, UK), general practitioner offices (n=9, the Netherlands) and long-term care facilities (n=2, the Netherlands). These centres will be recruited in the Leiden/the Hague region through the Extramural Leiden Academic Network and the University Network Care sector Zuid Holland. Data will be collected between August 2024 and August 2026. The study protocol was reviewed by the local research committee at Leiden University Medical Center (LUMC), who declared that the medical research involving human subject act (Dutch abbreviation: WMO) does not apply to the current study. On the 24 September 2024, the study was registered at clinicaltrial.gov (NCT06610721).

### Patient and public involvement statement

In this study, a patient panel specifically focused on UTIs was first involved during the early stages of the study design. We invited panel members to provide feedback on the overall study and more specifically to the research question, outcome measures and feasibility of the study. They helped us identify potential barriers to participation on which the study design was adjusted. They were also involved in assessing the burden on patients and the time to participate. Regarding the dissemination of the study results, the patient panel will be involved in determining what and how information will be communicated to the public. Their involvement is essential in ensuring that the study takes the needs of patients living with UTIs into account.

### Study participants

In every participating healthcare setting, the patients will initially be approached by their attending physician and provided with the patient information sheet. The study team (research physician, research nurses) will subsequently execute the informed consent procedure and will be responsible for follow-up and data collection.

Patients aged 65 years and older, both male and female, are eligible if the treating healthcare team determines that there is a clinical indication to perform diagnostic testing for a UTI. Importantly, inclusion is aligned with the clinical decision point where a urinalysis would normally be performed to confirm or rule out an UTI.

Urinary tract abnormalities, urolithiases, autoimmune disease or any form of immune compromised status are not considered exclusion criteria. Similarly, patients who recently used antimicrobial or immunosuppressant therapy will not be excluded. These broad inclusion criteria reflect the current real-world use of urinary diagnostic testing in clinical practice. By minimising exclusions, we aim to ensure that the study population closely resembles the heterogeneous group of patients for whom these tests are routinely used. Patients with pre-existing decision-making incapacity (eg, due to advanced dementia) are excluded from participation. However, patients with temporary or reversible conditions affecting decision making (eg, delirium) are eligible if they provide informed consent during the course of the study (deferred informed consent procedure). Patients with an indwelling catheter will be excluded from the study.

### Data collection and measurements

At baseline, the patients’ demographic data, medical history, current symptoms and vital signs will be collected by a trained member of the research team. The 4 A’s test (4AT),^23^ Katz Index of independence in activities of daily living (ADL) tool^24^ and PROMIS global health scale (v1.2)^25^ will be used to measure patients’ cognitive state, independence in performing daily activities and overall health respectively.

A sterile urine container will be provided to the patient along with instructions on how to obtain a clean midstream urine sample. Subsequently, patients will be asked to provide a fresh urine sample on-site. The urine sample is processed by the laboratory as soon as possible after urine production but not later than 2 hours after urine production to ensure optimal quality for urinalysis. Follow-up via telephone and electronic patient record (EPR) will be performed at 1 and 8 weeks to collect duration of symptoms, clinical outcome, complications of the infection, hospital admission, recurrence of infection and quality of life (table 1).

**Table 1:** Inclusion procedure

**Variable**	Baseline	Week 1	Week 8
Symptoms	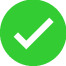	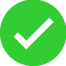	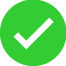
Medical history	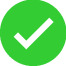	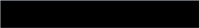	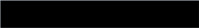
Medication use	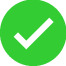	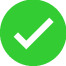	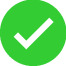
Vitals	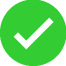	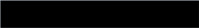	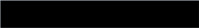
PROMIS global health scale	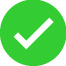	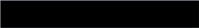	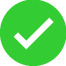
4AT	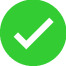	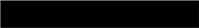	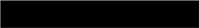
KATZ ADL scale	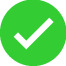	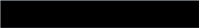	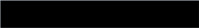
Urine sample	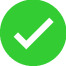	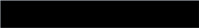	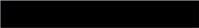
Complications	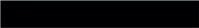	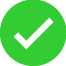	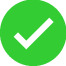
Hospital admission	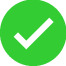	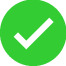	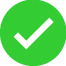
Recurrence of UTI <2 months	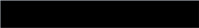	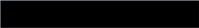	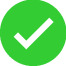

ADL, activities of daily living; 4AT, 4 A’s test; PROMIS, Patient-Reported Outcomes Measurment System ; UTI, urinary tract infection.

#### Sediment, culture and biomarker analyses

The collected urine will be divided into three portions after collection, one for urine sediment analysis, which includes leucocyte counts; one for urine culture; and one for biomarker analysis. For patients from participating long-term care facilities and general practitioners, all analyses will be performed in the LUMC in the Netherlands (Department of Medical Microbiology and Department of Laboratory Medicine and Clinical Chemistry). For patients presenting in participating hospitals, routine urine sediment analysis and urine culture will be performed on site, while urine for biomarker analysis will be stored locally by −70°C until biomarker analyses will be performed in batch in the LUMC.

Pyuria will be quantified by automated microscopy or flow cytometry (Sysmex UF-4000, Kobe; Japan).

For culture, 10 µL of non-centrifuged urine will be added to routine culture media and incubated overnight. A positive culture will be reported in colony forming units/mL (CFU/mL) and classified as a monoculture if ≥90% of the cultured colonies are from a single species.

For biomarker analyses, urine will be transferred into a 15 mL collection tube and centrifuged for 8 min at 3000 g at room temperature. The supernatant will be transferred into another collection tube and vortexed. The urine will be divided into six aliquots and stored at −70°C. Leftover samples will be stored frozen according to local LUMC protocol for 15 years, after which the samples will be destroyed.

Urine biomarkers will be analysed in batch using an in-house developed and validated multiplex liquid chromatography-mass spectrometry (LC-MS) and ELISA. LC-MS will be used for the biomarkers NGAL, TIMP-2 and CXCL-9, and ELISA for IL-6 and azurocidin.^22^

### Outcome measures

The primary outcomes of this study are the diagnostic accuracy of the individual urine biomarkers (NGAL, IL-6, AZU, TIMP 2, CXCL9), a combination of biomarkers and the pyuria cut-off level of 200 leukocytes/µL. This cut-off level is based on the results of the pilot study which showed that 200 leukocytes/µL resulted in specificity of 86% while maintaining a high sensitivity of 89%.^16^ For the definition of a UTI, a recently developed international reference standard will be used, whereby patients will be categorised as having a definite UTI, probable UTI, possible UTI or no UTI.^26^ This reference standard was developed in a worldwide Delphi procedure and consists of (1) local signs/symptoms including LUTS, (2) systemic signs and symptoms, (3) routine urinalysis results and (4) culture results. In this manner, a more accurate and standardised approach is used to minimise misclassification. To investigate the relationship between biomarker levels and disease severity and recurrence, the following secondary outcomes are chosen: the duration of symptoms in days, complications including bacteremia, sepsis, septic shock defined in accordance with the quick sequential organ failure assessment score, and renal or prostatic abscess confirmed by ultrasound or CT scan, lengths of hospital stay in days and the frequency of recurrence of UTI defined by international reference standard within 2 months after inclusion.

### Statistical analysis

#### Sample size

Power analyses are based on the results from the pilot study.^22^ For the biomarker panel, specificity was 91% at a sensitivity of 88%; however, power analysis was based on individual biomarkers to have an adequate sample size for assessment of both the individual markers and the panel. For individual biomarkers, sensitivity ranged from 76% to 87%, and specificity ranged from 72% to 95%. Lower bounds were used for the calculations, to ensure an adequate sample size. To assess specificity, with an α of 0.05, with (1 − α) % confidence level and a maximum marginal error of estimate of 0.10 (δ) for constructing CI of true value of specificity, assuming a value of 72% and using the normal approximation, and a disease prevalence of 30%, the total N needs to be 110. For sensitivity, using an assumption of sensitivity 76%, N would be 234.^27^ With an estimated drop-out of 5%, 246 patients need to be included.

#### Analysis of outcome measures

All statistical analyses will be performed using IBM SPSS V.29. Baseline characteristics will be summarised by standard descriptive statistics.

Areas under the receiver operating characteristics (ROC) curves for identifying UTI will be calculated to assess the diagnostic power of the individual biomarkers. For each biomarker, the optimal cut-off will be determined based on the ROC curves, and the associated sensitivity and specificity, positive and negative predictive values, and positive and negative likelihood ratios will be reported. An additional logistic regression analysis will be performed to assess the value of combination(s) of biomarkers, aiming at a minimal sensitivity and specificity of 80%. For urine leukocytes, the above-mentioned parameters will be reported for a cut-off of 200 leukocytes/µL.

Subgroup analyses will be performed to assess the performance of biomarkers in males and females, different settings (hospital, primary care setting and long-term care) and different clinical presentations (systemic symptoms vs localised symptoms). Categorical values will be compared using the Fisher exact test or Pearson χ2 test.

Additional comparative analyses will be performed to compare biomarker levels in UTI with and without systemic symptoms. To assess the correlation between biomarker levels and time to resolution of symptoms and length of hospital stay, Pearson’s correlation coefficient will be used. For the calculation of the association between biomarker levels and complications of UTI and recurrence of UTI, logistic regression will be used.

### Ethics and dissemination

This study protocol has received formal approval from the scientific committee of the LUMC in the Netherlands. The protocol was also reviewed by the local research (nWMO) committee, who declared on 15 April 2024 that the medical research involving human subject act (Dutch abbreviation: WMO) does not apply to the current study (reference number nWMODIV2_2024025). On 6 December 2024, the study received approval from the NHS Research Ethics Committee in the UK (reference number 24/LO/0649).

The dataset generated during this study will be pseudonymised and will only be available from the corresponding author on reasonable request.

We plan to publish the study findings in a peer-reviewed journal and present them at academic conferences. Additionally, the results will be disseminated to healthcare providers in both primary and secondary healthcare settings.

## Discussion

The UTI-GOLD is an observational multicentre diagnostic study with the aim to validate biomarkers for an accurate diagnosis of UTI in older people. A better diagnostic test is highly needed to improve timely and appropriate treatment of UTI in older adults and avoid the negative consequences of over- and underdiagnosis.

### Reflection on study design

One of the key strengths of this study is its performance across different real-life healthcare settings, characterised by broad inclusion criteria and recruitment from the three primary locations where UTIs are diagnosed: general practice, long-term care facilities and hospitals. This approach enhances validity and the applicability of the results to a wide population of older adults.

One of the largest challenges in all UTI studies, including the UTI-GOLD study, is the risk of misclassification due to the atypical presentation of older people and the lack of a gold standard for diagnosis. To minimise this risk, a recently developed consensus-based reference standard will be used to define UTIs. This standard was comprised by a national and international panel of UTI experts using a robust Delphi method, with a high degree of consensus. In the absence of a gold standard, this approach represents the most appropriate alternative to reduce classification bias and enhance homogeneity in UTI research.

The broadly defined inclusion criteria allow for the inclusion of patients with a wide variety of clinical presentations, comorbidities and frailty levels, representing a ‘real-world’ setting. Patients with an indwelling catheter were chosen to be excluded from the study since they are a separate patient population with a distinct pathophysiological mechanism and a different diagnostic approach. Similarly, patients with pre-existing decision-making incompetence will be excluded due to their inability to provide informed consent. Patients with delirium—a common symptom in older people with UTIs—will be eligible with a deferred informed consent procedure. This approach is essential, as patients with delirium represent the group in whom diagnostic uncertainty is greatest, and the use of a structured research reference standard enables their responsible inclusion in the study. It is important to acknowledge the unmet need for a reliable diagnostic tool to diagnose UTIs in patient populations that are excluded in the current study.

Another important consideration is the challenge of obtaining a midstream urine sample. While this is the preferred collection method to minimise contamination, it is challenging in the older population, especially in frail patients. While the study aims to collect optimal urine samples through clean-catch midstream collection, it also accepts samples that may not meet this standard. By doing so, the study avoids significant selection bias and supports the development of diagnostic tests that are not only scientifically robust but also applicable in real-world settings where ideal sample collection methods may not always be feasible.

### Future implications

Currently, there is no reliable diagnostic tool to diagnose UTI in older adults. To address this unmet need, a point-of-care (POC) test would be ideal, one that can be easily deployed across different healthcare settings, particularly outside the hospital. The UTI-GOLD study aims to identify the most appropriate biomarker(s) signature for a POC test. Once a POC test is developed, the test must be implemented in a study to evaluate its impact on patient outcomes in daily clinical practice.

With validated and reliable diagnostic markers for UTIs in older adults, timely and appropriate treatment can be improved, reducing the negative consequences of over- and underdiagnosis. The development of a POC test based on these markers could significantly contribute to antimicrobial stewardship. Consequently, such a test has the potential to positively impact patient outcomes, lower healthcare costs and revolutionise the care for patients with UTIs globally.
