# Supplementary material for: Validation of urinary biomarkers for accurate diagnosis of urinary tract infections in older adults across primary care, hospitals and long-term care facilities in the Netherlands and UK (UTI-GOLD): a multicentre observational study protocol
Source: BMJ Open. 2025 Jun 18;15(6):e103311. doi: 10.1136/bmjopen-2025-103311 (PMC12184357; doi:10.1136/bmjopen-2025-103311)
Supplement: Supplementary file 1 [file bmjopen-2025-103311.reviewer_comments.pdf]

PEER REVIEW HISTORY

BMJ Open publishes all reviews undertaken for accepted manuscripts. Reviewers are asked to complete a checklist review form and are provided with free text boxes to elaborate on their assessment. These free text comments are reproduced below.

ARTICLE DETAILS

Title (Provisional)

Validation of urinary biomarkers for accurate diagnosis of urinary tract infections in older adults across primary care, hospitals, and long-term care facilities in the Netherlands and United Kingdom (UTI-GOLD): A multicenter observational study protocol

Authors

El Moussaoui, Nora; van Andel, Esther; van der Beek, Martha T.; Vlot, Jessica; Bilsen, Manu; van Nieuwkoop, Cees; Lauw, Fanny; Delfos, Nathalie; Stalenhoef, Janneke E; Sijbom, Martijn; Akintola, Abimbola A.; Achterberg, Wilco P; Goeman, Jelle; Leyten, Eliane; van Uhm, Janneke; Corstjens, Paul L.A.M.; Mooijaart, Simon; Conroy, Simon; Cobbaert, Christa; Visser, Leo G.; Lambregts, Merel

---

This article was not externally reviewed at BMJ Open. Protocols that have been independently assessed prior to submission to BMJ Open are usually fast-tracked to publication on the grounds that further substantial changes will not be possible. This independent assessment will usually be external, independent review for both a substantial grant award from a non-commercial or government funder and ethics approval.
